# TEAL-Seq: targeted expression analysis sequencing

**DOI:** 10.1128/msphere.00984-24

**Published:** 2025-04-22

**Authors:** Georgia Doing, Priya Shanbhag, Isaac Bell, Sara Cassidy, Efthymios Motakis, Elizabeth Aiken, Julia Oh, Mark D. Adams

**Affiliations:** 1 The Jackson Laboratory for Genomic Medicine Farmington 481263 , Farmington, Connecticut, USA; University of Nebraska Medical Center College of Medicine, Omaha, Nebraska, USA

**Keywords:** metagenomics, targeted sequencing, skin microbiome

## Abstract

Metagenome sequencing enables the genetic characterization of complex microbial communities. However, determining the activity of isolates within a community presents several challenges, including the wide range of organismal and gene expression abundances, the presence of host RNA, and low microbial biomass at many sites. To address these limitations, we developed “targeted expression analysis sequencing” or TEAL-seq, enabling sensitive species-specific analyses of gene expression using highly multiplexed custom probe pools. For proof of concept, we targeted about 1,700 core and accessory genes of *Staphylococcus aureus* and *S. epidermidis*, two key species of the skin microbiome. Two targeting methods were applied to laboratory cultures and human nasal swab specimens. Both methods showed a high degree of specificity, with >90% reads on target, even in the presence of complex microbial or human background DNA/RNA. Targeting using molecular inversion probes demonstrated excellent correlation in inferred expression levels with bulk RNA-seq. Furthermore, we show that a linear pre-amplification step to increase the number of nucleic acids for analysis yielded consistent and predictable results when applied to complex samples and enabled profiling of expression from as little as 1 ng of total RNA. TEAL-seq is much less expensive than bulk metatranscriptomic profiling, enables detection across a greater dynamic range, and uses a strategy that is readily configurable for determining the transcriptional status of organisms in any microbial community.

**IMPORTANCE:**

The gene expression patterns of bacteria in microbial communities reflect their activity and interactions with other community members. Measuring gene expression in complex microbiome contexts is challenging, however, due to the large dynamic range of microbial abundances and transcript levels. Here we describe an approach to assessing gene expression for specific species of interest using highly multiplexed pools of targeting probes. We show that an isothermal amplification step enables the profiling of low biomass samples. TEAL-seq should be widely adaptable to the study of microbial activity in natural environments.

## INTRODUCTION

As microbiome research matures, studies are shifting from those that establish correlations between microbial profiles and disease states to those that define the mechanisms by which microbes impact host physiology (1, 2). Metagenome shotgun sequencing (mWGS) analysis has been a powerful discovery tool, but it does not address the functional state of constituent microbes. Measurement of microbial gene expression in a natural context is key to understanding the mechanistic forces driving commensalism and pathogenesis within community and host relationships (3–6).

Changes in diet, drug treatment, introduction of a pathogen, or alteration in host pathways may change the activity of the microbiome, not just its composition (6, 7). Metatranscriptome (metaTx) profiling can uncover which organisms are transcriptionally active and which genes are expressed. However, the efficacy of metaTx is limited by the large dynamic range of organismal abundance, gene expression levels, and—in some cases—the presence of host transcripts. Thus, quantitation of gene expression in lower abundance organisms by untargeted metaTx sequencing remains infeasible due to the cost of sequencing to sufficient coverage depth. Recently published metaTx studies have illustrated this point. One study evaluated the contribution of vitamin B12 production by skin microbes to the development of acne (8). Another examined transcription of the fecal microbiome of adult men (9). Both studies required ~100 million reads/sample. New, more accessible methods are needed to transcriptionally profile microbes of interest in their natural milieu.

The skin is an ideal proving ground for the development of new technologies that measure microbial gene expression in mixed communities. The abundance of bacterial species can vary widely at different skin sites, but importantly, abundance is a poor prognosticator of functional importance (10–12). At most skin sites, host cells contribute over 90% of the total extracted nucleic acids, making mWGS and metaTx extremely inefficient, particularly for characterizing lower abundance organisms (13). *Staphylococcus (S*.) spp. are keystone contributors to cutaneous immunity, barrier integrity, and microbial community homeostasis, including antagonism with skin pathogen *S. aureus* (14–17). Our previous metaTx work with skin swabs, however, yielded <5% of reads corresponding to microbial transcripts, highlighting the challenges of studying these communities using common methods (unpublished; see Table S4).

Targeting microbial profiling to specific genes or genomes enables a more cost-effective and comprehensive evaluation of microbial composition and activity. By limiting the sequencing to targeted regions, information on low-abundance species that would otherwise require deep metaTx can be captured at a fraction of the cost, on par with bulk RNA-seq of isolated strains. Array-based (18–20) and capture-based (21–24) strategies have been used to assess bacterial and viral abundance, but those methods are difficult to customize, expensive, laborious, or all three. The availability of low-cost oligonucleotide pools from multiple vendors offers assay development options based on highly multiplexed custom-designed probes, including the ability to iteratively optimize pools based on performance and facile incorporation of new content.

We previously developed MA-GenTA (microbial abundances from genome tagged analysis) as a quantitative and cost-effective method for species-level microbial profiling (25). We used highly multiplexed (>16,000) oligonucleotide probes to derive relative abundance data for microbes from the mouse gut. MA-GenTA and mWGS data demonstrated excellent correlation down to 0.01% relative abundance (25) and enabled inference of gene pathway composition at a cost only modestly higher than 16S rRNA amplicon sequencing. Here we build on this experience by extending the methods to targeted gene expression profiling designated as TEAL-seq (targeted expression analysis sequencing), which provides cost-effective mRNA profiling when metaTx is not feasible.

## RESULTS

### Experimental design

We selected *Staphylococcus aureus* (*Sa*) and *S. epidermidis* (*Se*) for developing targeted transcriptome profiling as they are keystone species in the skin microbiome, important for human health and disease, and well-characterized genomically and functionally (26–28). Two targeting strategies were pursued: a single-primer extension (SPE) and a molecular inversion probe (MIP) method, previously published for high-plex SNP genotyping assays (29). SPE uses a single 40-base primer that anneals to target sequences to prime DNA synthesis on a partially constructed Illumina sequencing library. MIP involves hybridization of probes to targets, followed by extension across the gap regions, ligation, and amplification of closed circles using primers with Illumina-compatible tails. Both methods target a cDNA or genomic DNA template.

**Fig 1:**
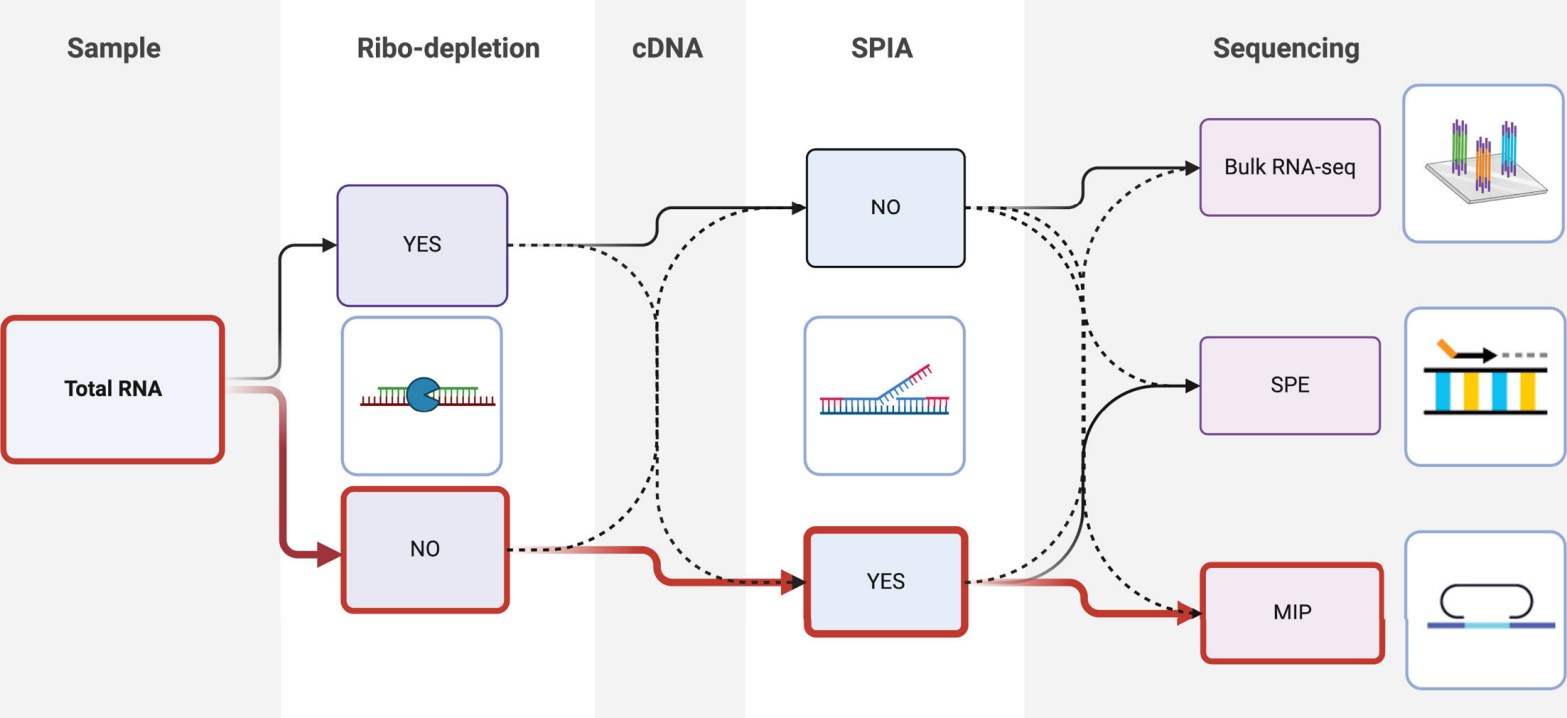
Layout of the experimental design. The experimental variables used for evaluating the targeted RNA-seq approaches are shown, illustrating how RNA samples were processed for analysis to determine the optimal TEAL-seq method, outlined in red.

We evaluated each targeted sequencing approach using RNA and genomic DNA isolated from pure cultures of *Se, Sa*, mixtures of the two, and controls that did not contain target sequences. We compared technical reproducibility, sensitivity to RNA input amount, specificity of targeting in mixed samples, the impact of rRNA depletion, the effect of pre-amplification of cDNA prior to capture, and compared targeted RNA-seq methods with standard bulk RNA-seq (Fig. 1).

### Probe pools yield >94% on-target and species-specific sequences

**Fig 2:**
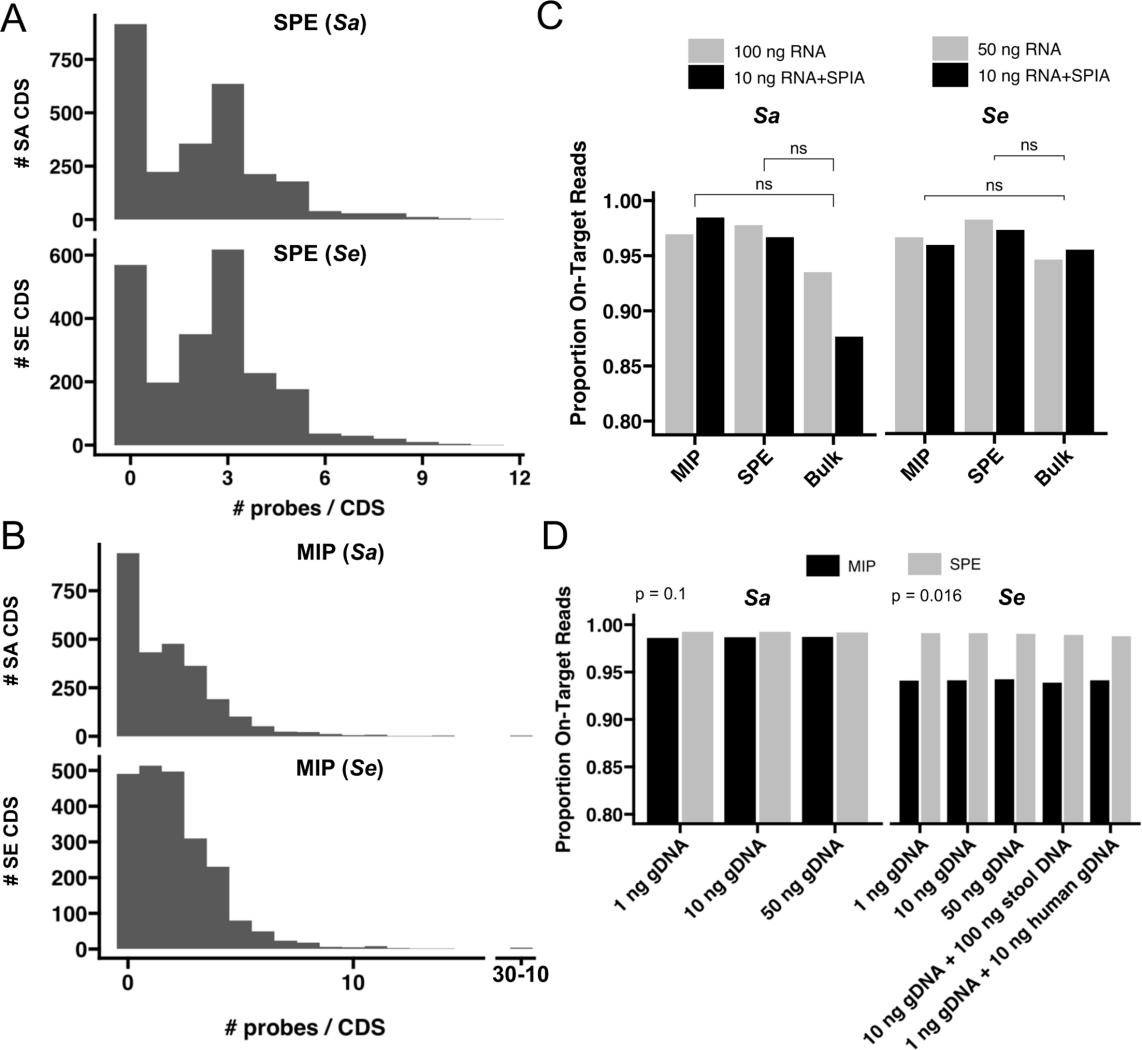
Distribution of the number of probes per gene in the targeted strains. (**A**) Probes target >50% *Sa* and *Se* CDS in SPE with at least one unique probe. (**B**) Probes target >50% *Sa* and *Se* CDS in MIP with at least one unique probe. (**C**) A high proportion of reads mapped on target for MIP, SPE, and Bulk RNA-seq without significant differences for RNA input amounts ranging from 10 ng to 100 ng as well as 1 ng with SPIA treatment. (**D**) A high proportion of reads mapped on target when tested on microbial gDNA controls and gDNA with obscuring mouse stool DNA or human gDNA.

The probe design for each species maximized within-species binding and limited cross-species binding (see Materials and Methods). We designed 6,121 SPE probes targeting 1,723 *Sa* coding sequences (CDS) and 5,879 SPE probes targeting 1,670 *Se* CDS (Fig. 2A). We also designed 4,960 MIP probes targeting 1,694 *Sa* CDS and 4,863 MIP probes targeting 1,748 *Se* CDS (Fig. 2B). A combined pool of *Se* and *Sa* probes was synthesized for each targeting method.

Given the low biomass of the microbiome at many body sites, we evaluated the use of single-primer isothermal amplification (SPIA) (30) to increase the amount of templates available for targeting and library construction. A theoretical advantage of targeted sequencing is that it makes depletion of rRNA unnecessary; we therefore also tested the effect of rRNA depletion on both bulk and targeted RNA-seq.

For both SPE and MIP, 94%–99% of reads map to the targeted regions across input RNA concentrations, indicating a high efficiency of targeting not significantly different from that of bulk RNA-seq (Fig. 2C, Table S2). Notably, no ribosomal RNA (rRNA) reads were detected, even in samples processed without rRNA depletion. Single-species experiments showed that probes were highly specific to the targeted genome when full-length reads were aligned to the reference genome with input amounts as low as 1 ng gDNA, which was used to assess performance independent of expression values. The high fraction of on-target reads was also observed for *Sa* and *Se* when input gDNA was mixed with mouse microbiome RNA (cDNA) or human gDNA (Fig. 2D). This indicates that cross-hybridization is minimal, even in the presence of complex non-targeted nucleic acids.

### Targeted transcriptomics approaches were found to be reproducible

**Fig 3:**
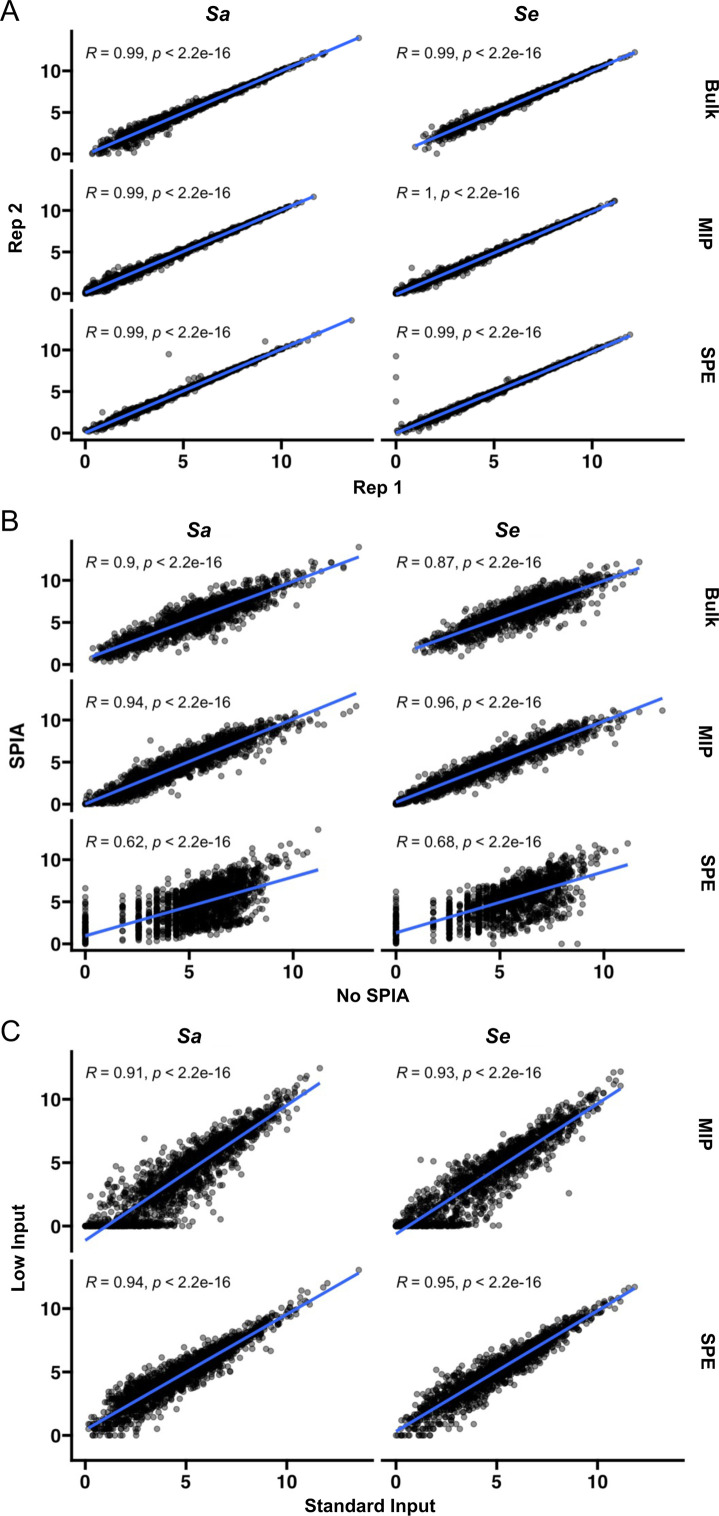
Evaluation of reproducibility using a 50:50 mixture of *Sa* and *Se* RNA. (**A**) Technical replicates (*n* = 2) are highly correlated for bulk RNA-seq, MIP, and SPE. (**B**) Samples with and without SPIA have a good correlation for bulk RNA-seq and MIP, but less so for SPE. (**C**) Samples with low (1 ng) and standard (10 ng) input total RNA amounts correlate well in MIP and SPE. Correlation coefficients (*R*) and *P*-values from Pearson tests.

We tested the reproducibility of inferred gene expression levels based on counts per million (CPM) per probe using MIP and SPE by comparing variation across technical replicates. We found technical replicates (libraries prepared from the same RNA sample) highly correlated for all sequencing approaches (Fig. 3A). Subsequently, we determined that libraries from the same RNA sample with and without SPIA treatment were highly correlated for bulk RNA-seq and MIP, but less so for SPE (Fig. 3B). Furthermore, when SPIA was used, libraries prepared from standard amounts of RNA (“standard input,” 10 ng) and less RNA (“low input,” 1 ng) were still correlated for both MIP and SPE (Fig. 3C). The low amount of intra-method variation makes targeted transcriptomics a promising approach for measurement of mRNA abundances, and next we assessed the accuracy of such measurements with subsequent comparisons to bulk RNA-seq and a case study of differential expression analysis.

### Probe performance varied across CDS

**Fig 4:**
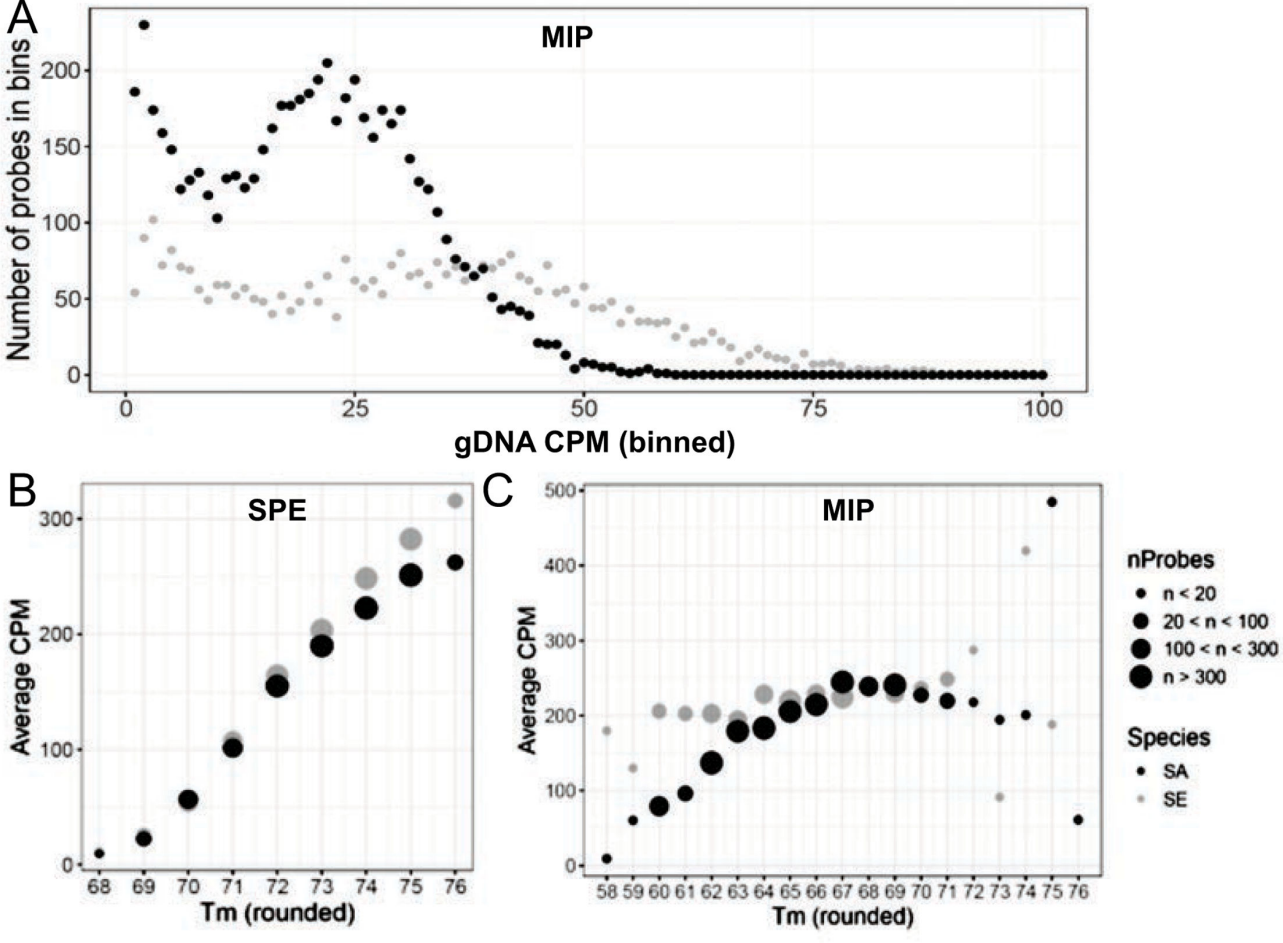
Probe performance varies and correlates with melting temperature in SPE data more so than MIP data. (**A**) Sequencing of genomic DNA with SPE shows a range of CPMs for *Sa* (black) and *Se* (gray) probes. (**B**) Probe CPM correlates with melting temperature in SPE data for both *Sa* (black) and *Se* (gray). (**C**) CPM has minimal correlation with melting temperature in MIP data for both *Sa* (black) and *Se* (gray).

Targeted sequencing of *Se* and *Sa* gDNA was used to evaluate the extent of variation in probe performance. We observed a wide range of CPM/probe, with *Se* probes having a much broader range than *Sa* probes in both the SPE and MIP data sets (Fig. 4A). Since *Se* and *Sa* probes were synthesized as a single pool, there is no *a priori* reason for the different performance between the two species. We considered whether there is a gradient of genomic copy number ranging from the origin to the terminus of replication due to differences in growth rate (31); however, we found that genome position was poorly correlated with CPM levels in both species (Fig. S1).

For SPE probes, there was a marked increase in average CPM with probe melting temperature (T_M_) (Fig. 4B), whereas T_M_ had minimal correlation with CPM for MIP probes (Fig. 4C). To explore the impact of potential strain-level nucleotide variation, we compared the performance of probes with mismatches to the test genome with that of perfect-match probes. This is an important consideration for profiling microbial communities because the exact sequence of the target genomes will often be unknown. SPE probes showed a substantial reduction in read counts with increasing numbers of mismatches, whereas MIP showed little or no effect of up to three mismatches (Fig. S2). Given the limitations of the SPE approach, we focused our remaining analyses on the MIP data sets.

### Impact of ribosomal RNA depletion and isothermal amplification

**Fig 5:**
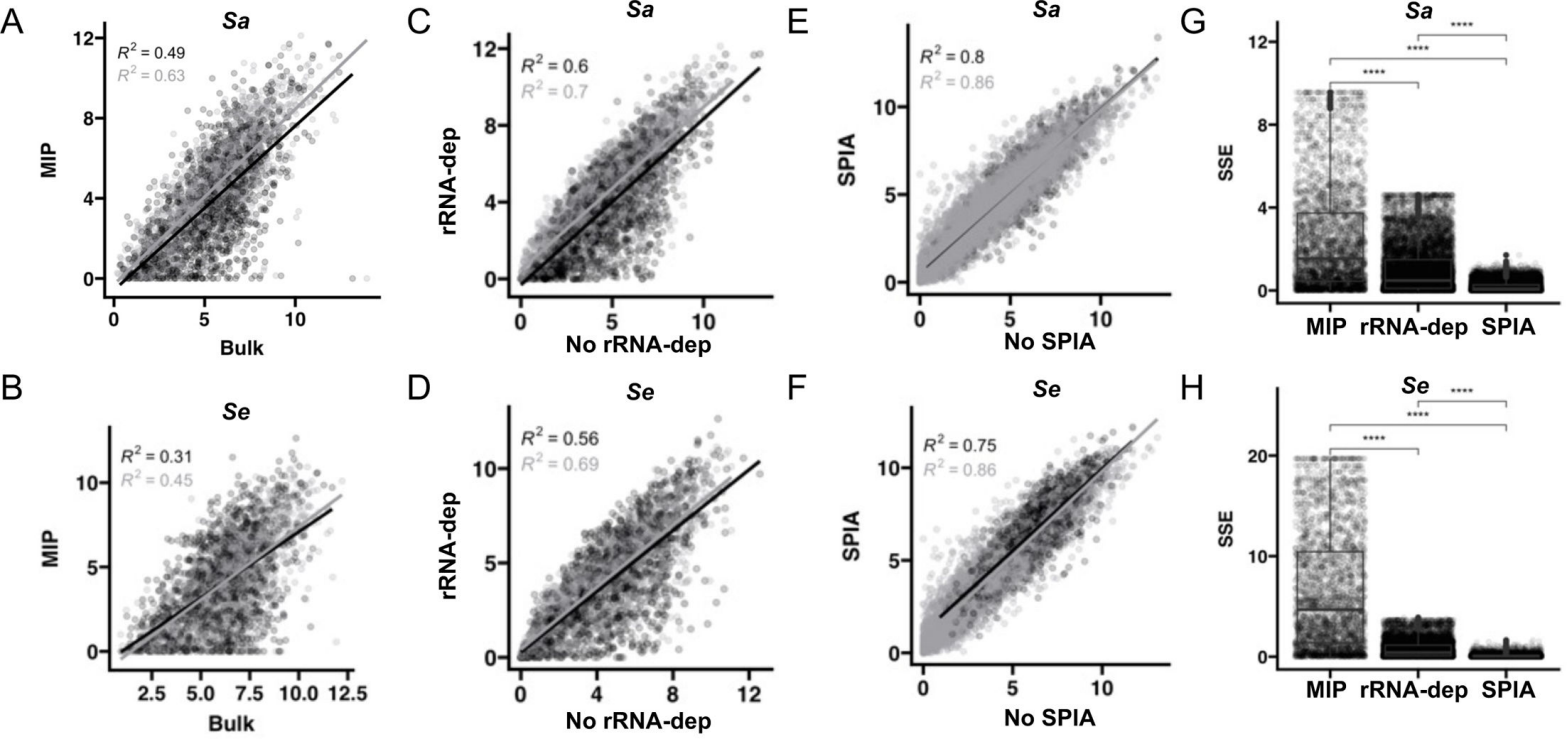
Sources of variation in targeted RNA-seq. (**A**) Comparison of CDS-level expression in bulk RNA-seq and MIP (targeted) without SPIA (black) and with SPIA (grey) for *Sa* and *Se.* (**B and C**) MIP with and without RNA depletion without SPIA (black) and with SPIA (gray) for *Sa* and *Se.* (**D and E**) MIP with and without SPIA amplification with ribo-depletion (black) and with ribo-depletion (gray) for *Sa* and *Se.* (**F and G**) Quantification of the sum of squared error (SSE) across comparisons for *Sa* and *Se.* (**H**) Correlation coefficients (*R*) and *P* values from Pearson tests. ****P < 0.0001, Wilcoxon test.

We tested the fidelity of targeting (MIP) with and without ribodepletion and compared the results to bulk RNA-seq. Given the variation in probe performances (Fig. 4), we expected a non-linear relationship in CDS-level gene expression inferred by MIP targeting and bulk RNA-seq and indeed found the two methods to correlate weakly (*R*^2^ = 0.31–0.63; Fig. 5A and B). Part of the difference could be explained by ribodepletion, a required step for bulk RNA-seq. The correlation in CDS-level expression levels as measured by MIP on RNA samples with and without rRNA depletion ranged from *R*^2^ = 0.56–0.70 (Fig. 5C and D). Thus, there is a substantial impact of ribodepletion on measurement of non-ribosomal transcripts that contributes to the low correlation between measurements collected via MIP and bulk RNA-seq.

To assess SPIA’s influence on mRNA measurements, we quantified mRNA abundance from bulk RNA-seq and MIP with and without SPIA. Bulk RNA-seq and MIP samples with and without SPIA were highly correlated (mean *R*^2^ = 0.83, Fig. 5E and F). Furthermore, CDS-level CPM values across bulk and targeted RNA-seq were less variable when SPIA amplification was used (Fig. 5A and B; gray) than when SPIA was not used (Fig. 5A and B; black). SPIA amplification introduces significantly less sum squared error (SSE) than ribo-depletion does (*P* < 0.0001 by Wilcoxon test) for both species (Fig. 5G and H), and we determined MIP with SPIA to be the optimal workflow for TEAL-seq (as outlined in Fig. 1). Probe-level variation combined with the effect of ribodepletion meant that targeted sequencing was unable to robustly replicate results from bulk RNA-seq; however, given its excellent reproducibility, we next aimed to evaluate the performance of TEAL-seq for differential expression analysis across conditions and for use in samples not amenable to bulk RNA-seq.

### TEAL-seq captured gene expression changes across growth conditions

**Fig 6:**
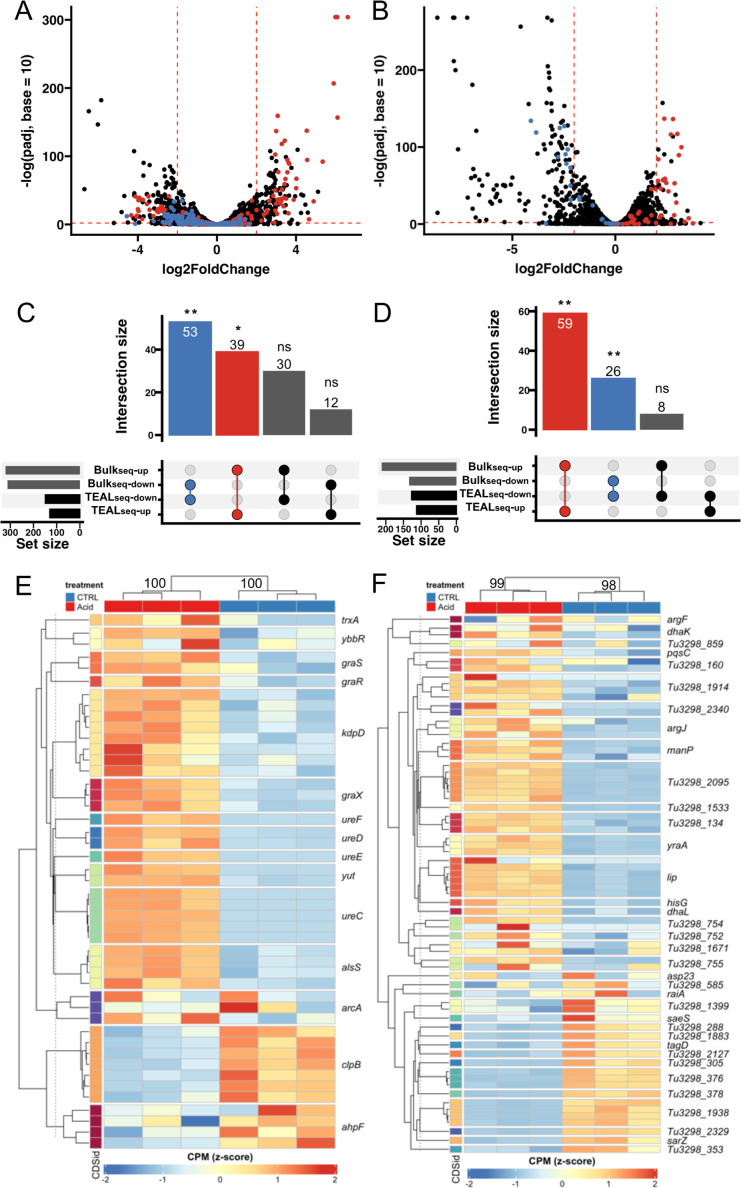
TEAL-seq detects acid stress responses consistent with previous studies using bulk RNA-seq. (**A**) Differential expression between TSB pH 7 and pH 4.8, as determined by |median probe log_2_FC| > 2 and median adjusted *P* value < 0.05 per gene in TEAL-seq, treatment shows upregulated (red) and downregulated (blue) regulated genes consistent with published bulk RNA-seq studies for *Sa* and *Se* (**B**) in similar conditions. In significant overrepresentation by hypergeometric test, 53 *Sa* genes were downregulated, and 39 *Sa* genes were upregulated by both bulk RNA-seq and TEAL-seq. Also in significant overlap, 59 *Se* genes were up-regulated and 26 *Se* genes were downregulated by both bulk RNA-seq and TEAL-seq. (**C**) Urease and acid response probes across conditions are consistent across CDSs for *Sa* and *Se*. Each column represents biological replicates (*n* = 3). (**D**) Sets of upregulated and downregulated acid response genes from TEAL-seq are over-represented by sets from published bulk RNA-seq studies of similar conditions. * adj. *P* < 0.05,** adj. *P* < 0.001 by hypergeometric test with Bonferroni correction.

RNA-seq analysis is often used to identify differentially expressed genes (DEGs) across conditions to infer underlying biology and nominate targets for follow-up studies. To test the ability of TEAL-seq to capture DEGs under physiologically relevant conditions, we measured gene expression from *Sa* and *Se* cultures grown in TSB (pH 7) and acidic TSB (pH 4.8) to model acid stress commonly encountered by staphylococci *in vivo*. TEAL-seq probes with the highest fold change (log_2_FC) in response to acid corresponded to genes that were consistent with known acid responses, in particular urease genes, as determined by previously published bulk RNA-seq studies for *Sa* (32) (Fig. 6A) and *Se* (33) (Fig. 6B). A stronger concordance in the *Se* data is not surprising; the bulk RNA-seq study used the same strain and pH as our study, whereas the *Sa* bulk RNA-seq study used slightly less acidic conditions (pH 5.5) and a different strain of *Sa*, JE2. Despite these differences, the most strongly upregulated and downregulated genes (|log_2_FC| > 2 and adjusted *P* value < 0.05 in bulk RNA-seq and |median probe log_2_FC| > 2 and |median probe adjusted *P* value| < 0.05 in TEAL-seq) were consistent for both *Sa* (Fig. 6C) and *Se* (Fig. 6D) showing significant enrichment of directionally specific gene sets (adjusted *P* < 0.05 by hypergeometric over-representation test with Bonferroni adjustment). Genes were determined to be differentially expressed in TEAL-seq using the absolute value of median fold change values of all probes targeting each gene to remain robust against outlier values from probes with poor performance. Furthermore, clustering of samples by genes identified via bulk RNA-seq showed that the TEAL-seq expression patterns (median probe CPM per gene for TEAL-seq data) were driven by condition for *Sa* (Fig. 6E) and *Se* (Fig. 6F), consistent with known facets of staphylococcal acid responses as reviewed in reference (34). Specifically, in *Sa*, TEAL-seq showed a strong increased expression of urease genes (*ureCDEF*, *yut*) (35), virulence factor regulators (*graSR*, *graX*) important for growth in acidic conditions (36, 37), and a pyruvate metabolism gene (*alsS*) that helps utilize protons to maintain intracellular pH (38). In addition, increases were observed in the expression of a gene involved in c-di-AMP regulation (*ybbR*), an osmolarity sensor (*kdp*), which participates in virulence factor regulation in acidic conditions (37), and a ROS-responsive thioredoxin (*trxA*) also deployed under acid stress (39). While the response of *Se* to acid stress is less well understood, TEAL-seq data showed an increase in the expression of arginine and acetone metabolism genes (*arjJ*, *dhaKL*), a cell membrane sensor (v*raS*), a lipase (*lip*), a mannose transporter (*manP*), and other genes found upregulated by bulk RNA-seq (33). Interestingly, some genes expected to increase in expression were found to decrease in expression in *Sa* (e.g., *clpB* and *ahpF*) and *Se* (e.g., *saeS*, *sarZ*, and *tagD*), which may be due to our analysis at only a single time point (late exponential growth, 8 hours). Overall, our results showed that TEAL-seq differential expression results were consistent with those obtained by bulk RNA-seq studies.

### Gene expression analysis from model and human samples

**Fig 7:**
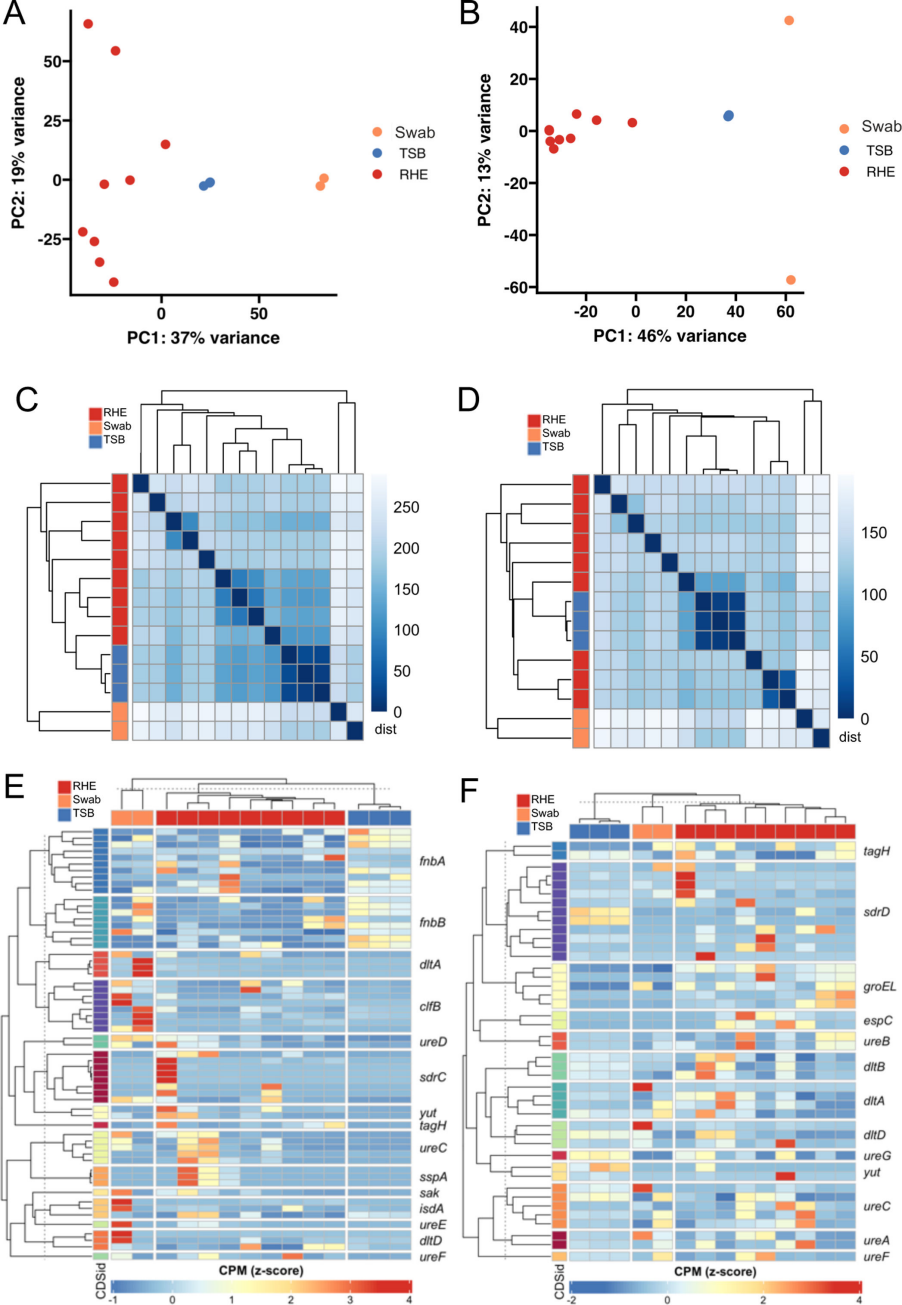
Genes previously implicated in colonization are heterogeneously upregulated in *Sa* and *Se* when grown on RHE and as sampled in nasal swabs. (**A**) PCAs separate expression from growth on RHE and expression from human nasal swabs from *in vitro* (TSB) in *Sa* and *Se* (**B**) disparately. (**C**) Sample correlations are driven by source *Sa* and *Se* (**D**) with different RHE samples forming subgroups. Values are pairwise distances in Pearson correlation matrices. (**E**) Genes important for colonization and urease genes are induced on RHE and in nasal swabs with substantial heterogeneity in *Sa* and *Se* (**F**).

To model the technical challenge of low amounts of material extractable from skin microbiomes, we applied TEAL-seq to (i) RNA extracted from the reconstructed human epidermis (RHE) colonized with clinical skin microbiome isolates and (ii) RNA extracted directly from human nasal swabs that were culture positive for staphylococci. *Sa* and *Se* transcripts were readily detected from both types of samples. Transcript levels derived from these sources were substantially different from cultures grown in TSB (PC1, separation by sample type accounted for 37% of the variance for *Sa* (Fig. 7A) and 46% of the variance for *Se* (Fig. 7B)). Notably, samples from independent RHE and swabs were more variable than samples from TSB, as expected for complex models, *ex vivo* samples, and clinical isolates. Furthermore, there was a high correlation in expression profiles for samples grown in TSB or between replicates of the same swab or RHE sample for both *Sa* (Fig. 7C) and *Se* (Fig. 7D). Thus, this variation between samples is likely biological due to differences in individual RHE specimens or genetic differences between the *Sa* and *Se* strains colonizing the nasal epithelium. The differences in mapping rates when we used reference genomes compared to isolate-specific genomes were insubstantial (Table S3), indicating that this approach is viable for the study of uncharacterized isolates. We attempted to compare results from TEAL-seq and bulk RNA-seq data on the same RHE sample; however, not all samples had detectable microbial mRNA using bulk RNA-seq, highlighting the need for targeted methods. Colonized RHE samples sequenced to depths of 8.5–42.1 million (mean = 30.4 million) contained only 0.06%–1.5% (mean = 0.3%) reads that mapped to *Sa* or *Se* mRNA (Table S4). Given the low return on microbial mRNA, we did not anticipate recovering sufficient reads to measure genome-wide expression and thus did not pursue further TEAL-seq to bulk RNA-seq comparisons on these samples.

We used TEAL-seq to examine expression patterns of genes described in the literature as important for staphylococcal colonization across RHE and nasal swab samples. A subset of nasal swabs and RHE samples showed increases in the expression of some colonization genes relative to TSB. For *Sa*, gene expression data from nasal swabs and RHE samples demonstrated increases in clumping factor B gene (*clfB*), staphylococcal antigen A (*isdA*), a teichoic acid modifier (*dltD*), and staphylokinase (*sak*), genes associated with biofilm formation and nasal colonization (40–42) (Fig. 7E), although these increases were variable across samples. *Se* gene expression exhibits a similar amount of heterogeneity, where some nasal swabs and RHE samples demonstrated increased expression of an extracellular protease (*espC*), a teichoic acid modifier (*tagH*, *dltABD*), and a stress-induced chaperone protein (*groEL*), all of which are implicated in colonization (43–46) (Fig. 7F). Other genes were surprisingly decreased or unchanged in expression across nasal swab and RHE samples compared to TSB, including fibronectin-binding proteins (*fnbAB*) in *Sa*. These data underline the need for more expansive studies to determine the mechanisms of nasal and RHE colonization; however, these preliminary data provide evidence that TEAL-seq is a useful method for such studies. Interestingly, urease genes display similar patterns to colonization genes in both species, suggesting swab and RHE samples may be taken from mildly acidic environments. These results demonstrate that TEAL-seq can be used to measure gene expression of *in vivo* relevant pathways in complex samples in a manner more sensitive than the current state-of-the-art method.

## DISCUSSION

We present an approach for targeted gene expression analysis optimized for samples with low biomass—TEAL-seq—that yields a high proportion of reads on target, even in the presence of complex background RNA or DNA. We tested two oligo-based methods for targeting, MIP and SPE, and determined MIP to be more robust than SPE. Both methods were capable of reproducibly inferring mRNA levels, but expression levels inferred from MIP had less probe-to-probe variation. Furthermore, we showed that cDNA amplification using SPIA reduced variation between replicates and had less impact on inferred expression level than rRNA depletion. The absolute correlation between TEAL-seq results and bulk RNA-seq was low, with contributions to noise from both probe-to-probe variation in TEAL-seq and the effects of ribodepletion in bulk RNA-seq. The cost of targeted sequencing is comparable to that of bulk RNA-seq when amplification is not needed, including SPIA raises the library prep cost but may be necessary when profiling low abundance microbes or low biomass samples that would otherwise be intractable with bulk RNA-seq (Table S1).

We showed that probes designed from genomes of laboratory strains captured gene expression information from other common experimental strains and clinical isolates of the same species. This is in part due to the short probe length (40 bases), ensuring coverage of conserved genes across strains, as well as the integration of data across multiple probes per CDS. Cross-strain capture is an important feature of this method, as the application of TEAL-seq to measure gene expression of real-world samples will require probes to be designed from existing genomic or metagenomic data. Our benchmarking of results from probes designed for *Sa* strain M2872 and *Se* strain ATCC14990 and applied to *Sa* strain USA300 and *Se* strain Tü3298 as well as clinical isolates of both species demonstrate the flexibility of TEAL-seq.

Differential expression analysis using TEAL-seq was able to recapitulate the biological signals observed previously using bulk RNA-seq. We used TEAL-seq to assess changes in gene expression of *Sa* and *Se* grown in TSB at pH 7 and pH 4.8 and identified highly concordant changes in expression compared to previous bulk RNA-seq studies. We confirmed that *Sa* responds to acid stress with urease genes, and *Se* does not. This is significant as urea is a human skin-produced metabolite that has important roles in barrier defense (47), and differences in *Sa* and *Se* responsiveness to acidic conditions may explain differences in invasiveness (48).

MetaTx has had limited usage in studying low-biomass microbiomes. However, TEAL-seq successfully captured predictable expression profiles from multiple complex sample types, including those isolated clinically. New approaches of this type are needed for the identification of population-level expression of virulence pathways or immunogenic metabolites at species resolution, as these will contextualize the function of the microbiome as a collective and refine our understanding of the contribution of individual species to the community (49–52). Additional studies applying TEAL-seq in the context of complex natural microbial communities will be needed to further demonstrate the potential of this method in experimentally relevant systems. However, the robust performance of TEAL-seq in capturing biologically significant transcriptional patterns from skin microbes grown under skin-relevant stress and reconstructed human epidermis makes it a promising tool for future applications.

## MATERIALS AND METHODS

### Bacterial samples and RNA and DNA isolation

Single colonies of *Staphylococcus aureus* USA300 and *Staphylococcus epidermidis* Tü3298 were inoculated into 2 mL Tryptic Soy broth (TSB) (BD cat:211825) and grown overnight at 37°C and 200 rpm agitation. Overnight cultures were sub-cultured into fresh 2 mL TSB medium at OD_600_ ~0.05 and allowed to grow to OD_600_ ~0.5–0.6. For stress conditions, as in reference 33, overnight cultures were grown as described above and then subcultured into mildly acidic TSB (pH 4.8) and allowed to grow to OD_600_ ~0.5–0.6. For RNA extraction, to 1 mL of culture, 2 mL of RNAprotect Bacteria Reagent (cat # 76506, Qiagen Inc.) was added, and cells were centrifuged at 8,000 × *g* for 5 minutes at 4°C. Cell pellets were then resuspended in RLT buffer (RNeasy Mini kit, cat # 74104, Qiagen Inc.) and transferred to 2 mL safe-lock tubes containing 50 mg acid-washed glass beads (150–600 µm diameter). Cells were homogenized using a TissueLyser for 4 minutes at 30 Hz/s and further processed according to the kit protocol. For DNA extraction, cells from 1 mL of culture were pelleted by centrifugation at 16,000 × *g* for 2 minutes, and DNA was extracted using the GenElute Bacterial Genomic DNA Kit (cat# NA2110, Sigma-Aldrich). *Escherichia coli* RNA was purchased from ThermoFisher (#AM7940). For mixed cultures, overnight cultures of individual strains were sub-cultured as described above, and equal numbers of cells from each strain were then inoculated into fresh 5 mL TSB at an OD_600_ of ~0.1–0.2 and grown at 37°C with agitation at 200 rpm for 2–3 hours until the OD_600_ reached ~0.5.

Mouse microbiome DNA was prepared as previously described (53). This study was carried out in accordance with the Guide for the Care and Use of Laboratory Animals and under the approval of the Institutional Animal Care and Use Committees at UCHC and JAX.

Nasal swab RNA was obtained and prepared as recently described (54). Briefly, samples were collected using sterile swabs (Puritan PurFlock Ultra, #22-029-454 506, Guilford, ME, USA). Two swabs were pre-moistened in nuclease-free water (Qiagen, Hilden, Germany) then inserted 2 cm into one nostril and rotated against the anterior nasal mucosa for up to 10 seconds. Nasal swabs were obtained from an approved biorepository, which was approved by the Jackson Laboratory Institutional Review Board.

### Reconstructed human epidermis cell culture cultivation

RHE tissue cultures were maintained as in Larson et al. (55), detailed methods provided in Supplemental Methods. RHE cultures were then dosed with 1.2 × 10^8^ CFU. Dosed RHE cultures were incubated for 1 hour at 37°C, then inoculum was aspirated and RHE were incubated with remaining bacteria for 18 hours before 200 µL of PBS (MatTek Corporation) was added to the apical surface, pipette mixed, and removed. 140 µL of RLT buffer +1% beta-mercaptoethanol was added to each RHE culture for RNA preservation. The RLT buffer-tissue solution was frozen at −80°C until RNA extraction.

### Target gene selection and probe design

Pangenome data sets for *Sa* and *Se* were assembled using Roary (56) based on the available complete genome sequences of each species: 51 complete *Sa* and 86 complete *Se* genomes downloaded from GenBank. CDS present in at least 50/86 *Se* and 30/51 *Sa* genomes were submitted for probe design. Candidate probe sets were designed using standard informatics workflows at Tecan Genomics for SPE probes and Molecular Loop for MIP probes. We selected probes for inclusion in the custom pool by subsampling based on perfect BLAST matches to at least 50 *Se* or 30 *Sa* genomes and exclusion of probes with alignments having fewer than five mismatches to the alternative species or a database of 10 *S*. *capitis* and 15 *S*. *hominis* genomes. All probes had <25 of 40 bases matching the human transcriptome, including rRNA genes. Additional filtering criteria removed probes at the extremes of melting temperature and sequence composition (requiring no runs of >6 of a single base) and established minimum probe spacing of at least 100 bases. All probes targeting both genomes were synthesized and used in a single pool. Distributions of the number of probes per CDS are provided in Fig. 2. Probe sequences are in Table S2. Purified nucleic acids were split across several workflows as shown in Fig. 1.

### Bulk RNA-seq library preparation and sequencing

The NEBNext Ultra II Directional Library Prep protocol (NEB #E7760S) was used with NEB’s bacterial rRNA depletion kit (NEB # E7850S) to make bulk RNA-seq libraries for whole transcriptome sequencing. 100 ng RNA input was used, and adaptors were diluted 25-fold before ligating to the cDNA. The same set of RNA samples was used in a hybrid workflow combining pre-amplification with the NEBNext Ultra II Library Prep. 10 ng of total RNA was subjected to rRNA depletion and then cDNA synthesis and amplification using the Single Primer Isothermal Amplification (SPIA) method with the Crescendo cDNA synthesis for qPCR kit (Tecan Genomics, Inc.). For these samples, RNA was not fragmented prior to cDNA synthesis. After SPIA amplification, cDNA was fragmented using components of the Allegro Targeted Genotyping V2 kit (Tecan Genomics, Inc.) before proceeding with end repair, adaptor ligation (25-fold diluted adaptors), and PCR-enrichment using the NEBNext Ultra II Directional Library Prep kit components. *E. coli* RNA was included as a positive control in both workflows. Index PCR was performed using the NEBNext Multiplex Oligos for Illumina (Dual Index Primers Set 1) (NEB #E7600S). The final library purification was carried out using 0.78× Ampure XP beads to remove the adaptor peak. Libraries were assessed on the TapeStation and Qubit dsDNA high-sensitivity assay before normalizing to 4 nM.

### cDNA synthesis and SPIA amplification

First- and second-strand cDNA syntheses were performed using the Crescendo cDNA synthesis for qPCR protocol (Tecan Genomics, Inc.). cDNA samples were then divided, with one portion subjected to SPIA amplification per the Crescendo protocol. cDNA was purified with AMPure XP beads at 1.8× and eluted in 33 μL of 1× TE. The resulting cDNA samples were quantified using NanoDrop following the kit protocol and Qubit dsDNA high-sensitivity assays. The samples that were amplified with SPIA yielded >1 μμg of cDNA, and the non-SPIA samples were 6–8 ng.

### Targeted RNA-seq library preparation and sequencing using single-primer extension probes

SPIA-amplified cDNA samples were diluted 20-fold, and 10–20 ng of cDNA was used as input for the Allegro Targeted Genotyping V2 protocol (Tecan Genomics, Inc.) with the custom probe pool. Non-amplified cDNA was not diluted, and ~1 ng was used as input for the Allegro protocol. Genomic DNA was used at 10–50 ng input. Each sample was enzymatically fragmented, followed by the ligation of barcoded adaptors. Barcoded samples were then purified, pooled, and placed in an overnight hybridization reaction mixture with the probe pool. The following day (>12 h), the DNA polymerase enzyme was added to the reaction for extension at 72°C for 10 min. Post-enrichment purification was done with 0.8× AMPure XP beads. A qPCR step was used to determine the number of cycles for library amplification (11 cycles). The final library pool was bead purified with 0.8× AMPure XP beads. A low template control (LTC) with 50 pg of *S. epidermidis* RNA input and a mouse stool metagenome DNA sample were used as sensitivity and specificity controls, respectively. The library pool was adjusted to 32 nM and 20 μL of the pool was used for sequencing (20 fmole/sample). The final sequencing pool also comprised libraries created using the NEBNext Ultra II Library prep protocol and Illumina DNA Prep (M) kit that were normalized to 4 nM and 0.2 nM, respectively, to represent 20 fmoles for the NEBNext samples and 1 fmole for the Illumina DNA Prep libraries. The exception was the *E. coli* RNA positive control (100 ng input) that was pooled at a lower amount to represent 5 fmoles in the sequencing pool. The library pool was loaded on NovaSeq 6000 SP flowcell 2 × 100 bp run. The custom read 1 (R1) primer provided in the Allegro kit was spiked into the Illumina read 1 primer position at 0.3 μM and a phiX174 control library was spiked in at 1% as recommended.

### Targeted RNA-seq library preparation and sequencing using molecular inversion probes

Targeted RNA libraries were prepared with the Low Input DNA Target Capture kit (Molecular Loop Biosciences, Inc.) using the cDNA generated with the Crescendo kit (Tecan Genomics, Inc.), including both the SPIA and non-SPIA pre-amplified samples, as well as the gDNA samples. The sample input was not normalized (50 pg–50 ng) to evaluate its effect on probe hybridization efficiencies and consequently the number of sequencing reads generated from each of these samples. Among the 32 samples, only the human blood high molecular weight genomic DNA sample was pre-treated by heating it to 95°C for 5 min for denaturation. The protocol was followed using the kit user guide, and the custom probe pool was hybridized for 18 hours (recommended ≥16 hours). After hybridization, each library was amplified by PCR for 20 cycles, pooled at equal volumes (10 μL each), and the library pool was purified with 0.8× Agencourt RNAXP Clean beads and eluted in 25 μL of 1×TE. The library pool was quantified with the Qubit high-sensitivity dsDNA assay and on the TapeStation D1000 high-sensitivity ScreenTape, which resulted in a single peak at ~415 bp. The library pool was loaded on a NovaSeq 6000 SP flowcell 2 × 100 bp run. The custom workflow was selected for the sequencing run using custom read1, read2, and sequencing primers provided by Molecular Loop. The phiX174 control library was spiked in at 1%.

### Illumina DNA Prep libraries

Libraries of *Se* and *Sa* gDNA were prepared using the Illumina DNA Prep, Tagmentation kit (Illumina, 20018705) and indexed using IDT for Illumina DNA/RNA UD Indexes (Illumina, 20042666). Reads were assembled using CLC Genomics Workbench, and the resulting assembly was compared to the reference sequences for the *Se* and *Sa* genomes, confirming that the cultured cells represented the expected genomes. Metadata and library sizes for all samples (bulk RNA-seq, MIP and SPE gDNA and RNA) are available in Table S5.

### Genome sequence alignment

Reads were processed with Trimmomatic 0.39 (57) to remove low-quality reads. For bulk RNA and SPE reads, the adapters were removed from the fastq reads using Cutadapt (58) version 4.4 s, using the bait sequence “AGATCGGAAGAG.” For MIP reads, seqtk v1.4-r122 was used (https://github.com/lh3/seqtk) to remove the five bases from the 5′ end of the sequences as *seqtk trimfq -b 5 < fastq* > . Reads were aligned to reference genomes *Sa* m2872 (GCF_017329165.1) and *Se* ATCC 14990 (GCF_006094375.1) as well as *Sa* USA300 (GCF_002993865.1) *Se* Tü3298 (GenBank record JBIQQI000000000.1, feature annotations available at https://github.com/ohlab/S.epi-CRISPRi-and-RNA-Seq) using BWA-MEM (59) version 0.7.17-r1188 as bwa mem -t 8 k 19 w 100. The mapped reads of each of the *Se* and *Sa* species were extracted with bamtools v2.5.2 (60) with parameter *-*isMapped *“*true*”* and subsequently filtered with MAPQ >30 and edit distance NM <4. High-quality sequences were sorted and converted into .bam and .bed files for downstream processing by SAMtools (61) v0.7.17-r1188. For bulk RNA-seq, the read count aligned to each CDS region was computed using featureCounts v1.6.4(62) (from Subread1.6.4) with -t CDS or -t rRNA, as appropriate, and summarized as counts per CDS per million reads per kilobase CDS length (RPKM).

### Probe sequence alignment

We also used BWA-MEM to align the SPE and MIP reads to a database of the probe sequences. For MIP, we used the seqinr R package (63) to extract the binding locations consisting of the first and the last 40 bps of the probe sequences and generated new fasta files with the “first 40 bps” and “last 40 bps” probe data. We aligned the reads of each sample separately to the probe sequences of each species. The mapped reads were extracted as before with bamtools v2.5.2 and subsequently filtered with MAPQ >30 and NM <1 (perfect match) in SPE and MAPQ >30 and NM <3 in MIP. The high-quality sequences were sorted and converted into bam and bed files for downstream processing by SAMtools v0.7.17-r1188. The raw counts were generated with *bedtools coverage* for each sample from the probe sequences (.bed) and cleaned read (.bam) files by bedtools v2.31.0 (64). The raw counts were CPM normalized as $1M\times r_{i}^{j,s}/R^{j,s}$, where $r_{i}^{j,s}$ denotes the number of reads mapped to location *i* in sample *j* and species *s*,$s\in \left\{Se,Sa\right\}$ and $R^{j,s}$ is the total number of mapped reads in sample *j* and species *s*. Probe count tables are provided as Table S6-S11.

### Differential expression analysis and correlation analyses

Differential expression analysis across stress conditions was performed in Rv4.4.1 (65) using DESeq2 (66) using a two-factor design matrix accounting for stress treatment (TSB or acid) and SPIA status including interaction terms. In all comparisons, differentially expressed genes were defined as those with fold change magnitude >4 and adjusted *P*-value < 0.01. Correlation matrices were calculated in R as Euclidean distances using the stats package (65), and pairwise correlations were assessed via Pearson correlation coefficients (*R* values) of best-fit linear models to two-dimensional data using ggpubr (67).

## Data Availability

All data used in these analyses are available at the NCBI Gene Expression Omnibus under accession GSE279187.

## SUPPLEMENTAL MATERIAL

Table S8AB msphere.00984-24-s0001.xlsx Counts table for MIP reads mapped to probe locations on the reference genome.

Table S9A msphere.00984-24-s0002.xlsx Counts table for MIP reads mapped to probe locations on the experimental genome.

Table S9CD msphere.00984-24-s0003.xlsx CDS-level counts for MIP reads mapped to the experimental genome.

Tables S1-S5 msphere.00984-24-s0004.xlsx Multiple supplemental tables.

Tables S6 and S7 msphere.00984-24-s0005.xlsx Bulk RNA-seq read counts and CPM/TPM values.

Supplemental material msphere.00984-24-s0006.docx Listing of supplemental materials, Figures S1 and S2, and supplemental methods.

Table S8CD msphere.00984-24-s0007.xlsx CDS-level counts for MIP reads mapped to the reference genome.

Table S9B msphere.00984-24-s0008.xlsx CPM tables for MIP reads mapped to the experimental genome.

Table S10 msphere.00984-24-s0009.xlsx Counts table for SPE reads mapped to the reference genome.

Table S11 msphere.00984-24-s0010.xlsx Counts table for SPE reads mapped to the experimental genome.

ASM does not own the copyrights to Supplemental Material that may be linked to, or accessed through, an article. The authors have granted ASM a non-exclusive, world-wide license to publish the Supplemental Material files. Please contact the corresponding author directly for reuse.
