# Supplementary material for: TEAL-Seq: targeted expression analysis sequencing
Source: mSphere. 2025 Apr 22;10(5):e00984-24. doi: 10.1128/msphere.00984-24 (PMC12108068; doi:10.1128/msphere.00984-24)
Supplement: Supplemental material — Listing of supplemental materials, Figures S1 and S2, and supplemental methods. [file msphere.00984-24-s0006.docx]

**Supplemental Materials List**

| **Supplemental Figure S1.** CPM bias based on gene position relative to origin of replication. | This document |
| --- | --- |
| **Supplemental Figure S2.** Read mapping to experimental genomes. | This document |
| **Supplemental Methods.** RHE cell culture. | This document |
| **Supplemental Table S1.** Estimated cost comparison**.** | mSphere00984-24-S0004.xlsx |
| **Supplemental Table S2**. Probe sequences. (A) Molecular Inversion Probe sequences used for *S. epidermidis*. (B) Molecular Inversion Probe sequences used for *S. aureus*. (C) Single Primer Extension probe sequences used for *S. epidermidis* and *S. aureus.* | mSphere00984-24-S0004.xlsx |
| **Supplemental Table S3.** Mapping statistics of probe targets captured with non-zero expression values using genomes from which probes were designed (S.a. M2872, S.e. ATCC 14990), genomes of the specific isolates used and experimental laboratory strains (S.a. USA300, S.e. Tu3298). | mSphere00984-24-S0004.xlsx |
| **Supplemental Table S4**. Read counts mapping to human and microbial references from bulk RNA-seq of select samples. | mSphere00984-24-S0004.xlsx |
| **Supplemental Table S5**. Sample metadata and library sizes for bulk RNA-seq, MIP and SPE. (A) Sample metadata. (B) Library sizes for bulk RNA-seq, MIP and SPE mapped to experimental genomes (expdb), *Sa* USA300 and *Se* Tü3398. (C) Library sizes for bulk RNA-seq, MIP and SPE mapped to reference probe design genomes (refdb), *Sa* M2872 and *Se* ATCC 14990. | mSphere00984-24-S0004.xlsx |
| **Supplemental Table S6.** Count table for bulk RNA-seq data in which reads are mapped to reference genomes from which MIP and SPE probes were designed (S.a. M2872, S.e. ATCC 14990). (A) Raw counts of bulk RNA-seq reads mapped to reference genomes. (B) Counts per million (CPM) of bulk RNA-seq reads mapped to reference genomes. (C) Transcripts per million (TPM) of bulk RNA-seq reads mapped to reference genomes. | mSphere00984-24-S0005.xlsx |
| **Supplemental Table 7.** Count table for bulk RNA-seq in which reads are mapped to the genomes of the strains with which experiments were conducted (S.a. USA300, S.e. Tu3298). (A) Raw counts of bulk RNA-seq reads mapped to experimental genomes. (B) Counts per million (CPM) of bulk RNA-seq reads mapped to experimental genomes. (C) Transcripts per million (TPM) of bulk RNA-seq reads mapped to experimental genomes. | mSphere00984-24-S0005.xlsx |
| **Supplemental Table S8.** Count table for targeted RNA-seq data using Molecular Inversion Probes (MIP) in which reads are mapped to reference genomes from which MIP and SPE probes were designed (S.a. M2872, S.e. ATCC 14990). (A) Raw probe counts of MIP data mapped to reference genomes from which probes were designed (S.a. M2872, S.e. ATCC 14990). (B) Probe counts per million (CPM) of MIP data mapped to reference genomes from which probes were designed (S.a. M2872, S.e. ATCC 14990). (C) Gene-level counts (median raw counts of probes for each CDS) of MIP data mapped to reference genomes from which probes were designed (S.a. M2872, S.e. ATCC 14990). (D) Gene-level counts per million (median CPM of probes for each CDS) of MIP data mapped to reference genomes from which probes were designed (S.a. M2872, S.e. ATCC 14990). | mSphere00984-24-S0001.xlsx  mSphere00984-24-S0007.xlsx |
| **Supplemental Table S9.** Count table targeted RNA-seq data using Molecular Inversion Probes (MIP) in which reads are mapped to the genomes of the strains with which experiments were conducted (S.a. USA300, S.e. Tu3298). (A) Raw probe counts of MIP data mapped to genomes of the strains with which experiments were conducted (S.a. USA300, S.e. Tu3298). (B) Probe counts per million (CPM) of MIP data mapped to genomes of the strains with which experiments were conducted (S.a. USA300, S.e. Tu3298). (C) Gene-level counts (median raw counts of probes for each CDS) of MIP data mapped to genomes of the strains with which experiments were conducted (S.a. USA300, S.e. Tu3298). (D) Gene-level counts per million (median CPM of probes for each CDS) of MIP data mapped to genomes of the strains with which experiments were conducted (S.a. USA300, S.e. Tu3298). | mSphere00984-24-S0002.xlsx  mSphere00984-24-S0003.xlsx  mSphere00984-24-S0008.xlsx |
| **Supplemental Table S10.** Count table for targeted RNA-seq data using Single Primer Extension (SPE) in which reads are mapped to reference genomes from which MIP and SPE probes were designed (S.a. M2872, S.e. ATCC 14990). (A) Raw probe counts of SPE data mapped to reference genomes from which probes were designed (S.a. M2872, S.e. ATCC 14990). (B) Probe counts per million (CPM) of SPE data mapped to reference genomes from which probes were designed (S.a. M2872, S.e. ATCC 14990). (C) Gene-level counts (median raw counts of probes for each CDS) of SPE data mapped to reference genomes from which probes were designed (S.a. M2872, S.e. ATCC 14990). (D) Gene-level counts per million (median CPM of probes for each CDS) of SPE data mapped to reference genomes from which probes were designed (S.a. M2872, S.e. ATCC 14990). | mSphere00984-24-S0009.xlsx |
| **Supplemental Table S11.** Count table targeted RNA-seq data using Single Primer Extension (SPE) in which reads are mapped to the genomes of the strains with which experiments were conducted (S.a. USA300, S.e. Tu3298). (A) Raw probe counts of SPE data mapped to genomes of the strains with which experiments were conducted (S.a. USA300, S.e. Tu3298). (B) Probe counts per million (CPM) of SPE data mapped to genomes of the strains with which experiments were conducted (S.a. USA300, S.e. Tu3298). (C) Gene-level counts (median raw counts of probes for each CDS) of SPE data mapped to genomes of the strains with which experiments were conducted (S.a. USA300, S.e. Tu3298). (D) Gene-level counts per million (median CPM of probes for each CDS) of SPE data mapped to genomes of the strains with which experiments were conducted (S.a. USA300, S.e. Tu3298). | mSphere00984-24-S0010.xlsx |


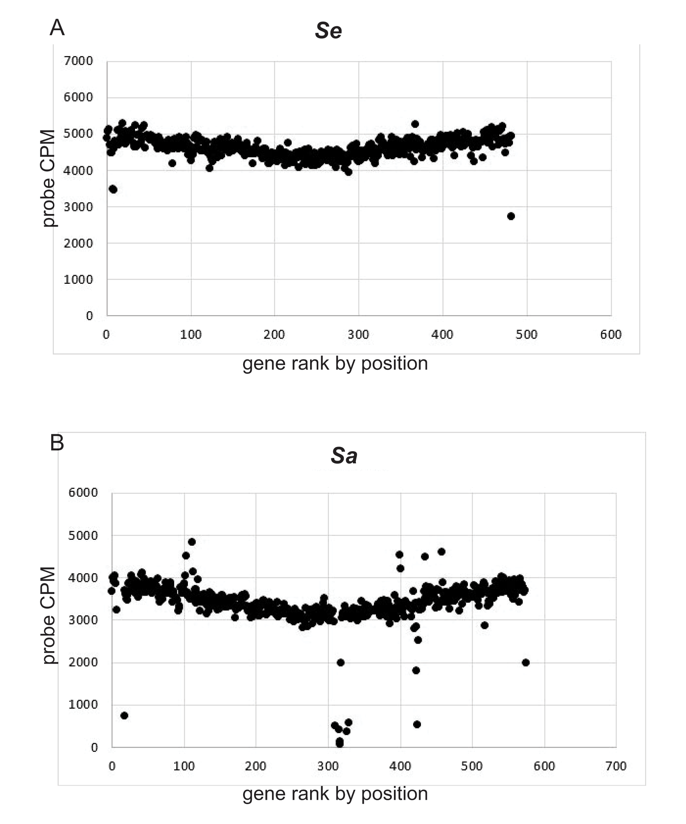


**Supplementary Figure S1. CPM bias based on gene position relative to origin of replication.** There is no substantial bias introduced by position relative to the origin of replication for *Se* Tü3298 (A) or *Sa* USA300 (B).


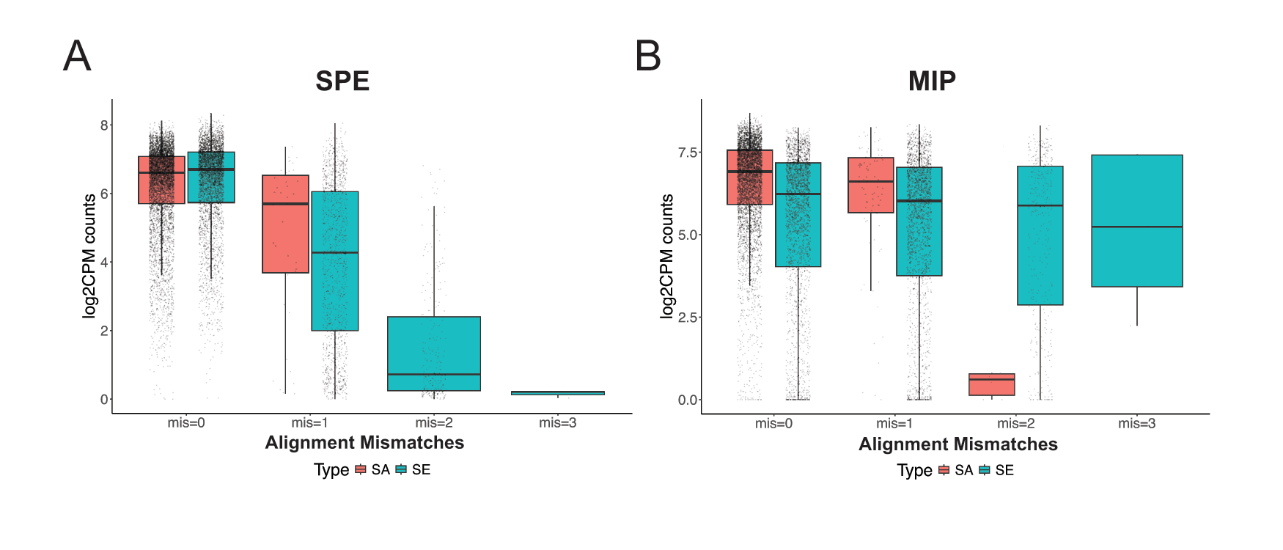
**Supplemental Figure S2. Read mapping to experimental genomes.** Mapping rates as the number of mismatches between the probe design sequence and the experimental genome increase for SPE (A) and MIP (B).

**Supplemental Methods**

**Reconstructed human epidermis cell culture cultivation.** 9mm primary normal human RHE tissue cultures were obtained from MatTek Corporation (EpiDerm, Gothenburg, Sweden). All batches were grown using cells from MatTek Corporation’s EpiDerm standard donor, a healthy male. The RHE were cultured according to the manufacturer’s directions. Briefly, upon arrival, the RHE cultures were placed in 6-well plates with 1mL of warmed antibiotic-free EpiDerm Maintenance Media (MatTek Corporation,) or the equivalent EpiDerm Assay Media basally per well. The basal media was replaced daily, and the RHE cultures were kept at 37°C with 5% CO_2_.

For each microbial isolate, a single colony was grown overnight in sterile 1X TSB. 10^8^ colony-forming units from each liquid culture were washed with ultrapure water (Fisher Scientific, #AAJ71786AP, Hampton, NH) and resuspended to a final concentration of 10^7^ colony-forming units in 120 µL.

The RHE cultures were then dosed with 120uL of microbial isolate or vehicle (ultrapure water alone). Dosed RHE cultures were incubated for 1 hour at 37°C, then inoculum was aspirated to restore the air liquid interface. RHE were incubated with remaining bacteria for 18 hours prior to harvest.

At harvest, 200 µL of PBS (MatTek Corporation) was added to the apical surface of each RHE culture, pipette mixed, removed, and plated for CFU enumeration. 140 µL of RLT buffer + 1% beta-mercaptoethanol was added to each RHE culture for RNA preservation. The RLT buffer-tissue solution was frozen at -80°C until RNA extraction. *S*. *epidermidis* Tü3298-GFP colonized RHE were visualized under blue light.
